# Optimisation of A Sample Preparation Method for the Determination of Multi-Elemental Compositions in Human Hair By Triple Quadrupole ICP-MS Analysis

**DOI:** 10.1007/s12011-025-04968-5

**Published:** 2026-01-20

**Authors:** Agneta Annika Runkel, Marta Jagodic Hudobivnik, Igor Živković, Polona Klemenčič, Darja Mazej, Milena Horvat

**Affiliations:** 1 Jožef Stefan Institute, Jamova cesta 39, Ljubljana, 1000 Slovenia; 2 Jožef Stefan International Postgraduate School, Jamova cesta 39, Ljubljana, 1000 Slovenia; 3 Division of Occupational and Environmental Medicine, Lund University, Scheelevägen 8, Lund, 22363 Sweden

**Keywords:** Human biomonitoring, Hair analysis, Multi-elemental analysis, ICP-MS/MS, Method validation

## Abstract

Monitoring toxic elements has a long tradition in Slovenia due to historical mining. More recently, attention has shifted to essential elements, since both deficiencies and excesses can harm health. Regular monitoring of (non-)essential elements supports risk assessment and policymaking. While urine and blood are common biomonitoring matrices, hair offers a non-invasive alternative that reflects exposure over several months, though standardised methodologies for hair analysis remain limited. This study aimed to develop and validate a sensitive and robust analytical method for the determination of 29 elements in human hair, addressing key challenges in sample preparation and contamination control. We developed a sensitive and robust method for the determination of 29 elements (Ag, Al, As, Ba, Ca, Cd, Co, Cr, Cu, Fe, Hg, K, Mg, Mn, Mo, Na, Ni, P, Pb, Rb, S, Sb, Se, Sn, Sr, Ti, U, V, and Zn) in 3 cm segments of human hair that involves a washing procedure with acetone and Milli-Q water, microwave digestion with 65% HNO_3_, and analysis with Triple Quadrupole Inductively Coupled Plasma Mass Spectrometry (ICP-MS/MS). Evaluation of preparation steps revealed stainless-steel scissors as a major contamination source. Glass digestion vessels were unsuitable for several elements due to high detection limits and relative standard deviations. The optimised method reduced analytical variability and improved sensitivity compared to published protocols. This validated method enables reproducible multi-elemental analysis in hair, highlights overlooked contamination risks, and is now applied in human biomonitoring studies to strengthen exposure assessment and standardisation efforts.

**Supplementary Information:**

The online version contains supplementary material available at 10.1007/s12011-025-04968-5.

## Introduction

Humans are continuously exposed to thousands of chemicals from the time of conception until death [1]. Human biomonitoring (HBM) is an essential method in assessing exposures in populations, with implications for risk assessment and political decision making [2]. In 2007, Slovenia initiated the first national human biomonitoring (HBM) survey to assess exposure to harmful compounds as stated in the national legislation (Act for Chemicals, No. 110/03). Since the beginning, trace elements have been of particular interest for monitoring, as Slovenia has two former mining sites that contaminated the local surroundings with lead (Pb) and zinc (Zn) in the Upper Mežica Valley and with mercury (Hg) in Idrija [3, 4]. Additionally, industrial activities in the towns of Celje and Jesenice are potential sources of elemental contamination [5]. While environmental monitoring after confirmed or suspected contamination is a common strategy for political decision-making and risk assessment in many countries, monitoring of essential elements provides valuable insights into the nutritional status of participants [6].

The most common matrices for the monitoring of trace elements in HBM studies are urine and blood, which both come with benefits and disadvantages [7]. The apparent advantages of urine sampling in population studies lie in the non-invasiveness and large acquisition volumes compared to blood [8]. However, the usefulness of a matrix highly depends on the research hypothesis or the aim of the study. For some elements, the matrix reflects different exposure scenarios; for instance, cadmium (Cd) in blood has a half-life of approximately three to four months, making it a suitable biomarker of recent exposure. In contrast, Cd accumulates in the kidneys, where it has a half-life exceeding ten years and is excreted only slowly, making urine a more appropriate matrix for monitoring long-term Cd exposure [9]. Therefore, while each matrix holds important information, the complete picture sometimes requires analysis from multiple angles. Human hair can provide information on long-term exposure, and segmental analyses can be used to reconstruct the history of exposure to, e.g., methylmercury and arsenic [8]. At the average growth rate of human scalp hair of 1 cm per month (Murphrey et al., 2022), hair analysis allows the analyst to choose the approximate exposure period prior to sampling. The possibilities are limited by the limits of detection (LOD) and quantification (LOQ) of the respective method, but studies report results from hair segments from 0.5 cm up to the full length [10, 11]. In HBM, the availability of hair segments presents another limitation, as participants might wear their scalp hair shorter than 3 cm. Therefore, exposure period, LOD, LOQ, and availability of hair samples are important factors to consider in the planning stage of HBM using human hair. Despite these considerations, it provides further advantages over blood and urine. It is easily collected and usually contains higher levels of contaminants than blood and urine [12]. However, to date, there is no scientific consensus on the correlation between concentrations of elements in hair and exposure, nor on the ability of hair analysis to give information on the body burden [12]. As such, it is understandable that knowledge of the toxicokinetics of the metals of interest is a crucial asset in the interpretation of element concentrations in various matrices, and this relies heavily on sensitive and robust analytical methods. These methods, while generally available, lack standardisation and a unified approach [13] and most studies neglect to evaluate the risk of contamination from sample handling materials and the affinity of some elements towards the materials of the digestion vessel or the digestion solution [10, 11, 14–18].

With this manuscript, we aim to facilitate standardization by presenting a sensitive and robust method for the determination of Ag, Al, As, Ba, Ca, Cd, Co, Cr, Cu, Fe, Hg, K, Mg, Mn, Mo, Na, Ni, P, Pb, Rb, S, Sb, Se, Sn, Sr, Ti, U, V, and Zn in 3-cm segments of human hair.

## Materials and Methods

### Consumables

Acetone for the washing of hair samples was purchased from Supelco, EMSURE^®^ ACS, ISO, Reag. Ph Eur and Milli-Q was obtained using an installed Milli-Q system (Merck, Millipore, Darmstadt, Germany). 65% HNO_3_, used for the digestion of hair samples, was purchased from Suprapur^®^ for trace analysis (Supelco^®^). Two certified reference materials (CRMs) were used: NIES No. 13 human hair and IAEA-086 Btl. No. 1995/658.

For the multi-elemental calibration curves, the following standard solutions and mixtures were used: Periodic table mix I for ICP (TraceCERT Merck Supelco^®^) and single-element standards for Hg (NIST SRM 3133), Ca, K, S, P, Mg, Mo, Sn, Sb, and Ti (Sigma Aldrich), Na (Merck Supelco^®^), and U (Fluka).

A single volunteer provided a hair sample that was divided into aliquots for the optimisation of the method for real hair samples. It will be referred to as “test hair samples” throughout the manuscript.

### Preparation of Working Solutions

A solution of 5% HNO_3_ was prepared by diluting 65% SupraPur HNO_3_ with Milli-Q water.

The 10 µg/g Periodic table mix I stock solution was used to prepare the calibration ranging from 0 ng/g to 250 ng/g by sequential dilution with 5% HNO_3_. Additional calibration points were prepared as mixtures of single-element standards for Na, Ca, K, S, P, and Mg by diluting the respective 1 mg/g stock solutions with 5% HNO₃ to obtain concentrations of 1, 2, 5, and 10 µg/g.

From single-element standards, mixtures (0 to 100 ng/g) were prepared for Mo, Sn, Sb, Ti, and U by diluting the respective stock solutions—1 mg/g for Mo, Sn, Sb, and Ti, and 10 µg/g for U—with 5% HNO₃.

For Hg, a separate calibration curve was prepared by diluting a 100 ng/g working solution of NIST SRM 3133 to obtain calibration points ranging from 10 ng/g to 0.1 ng/g in 5% HNO₃.

### Method Development

**Fig. 1:**
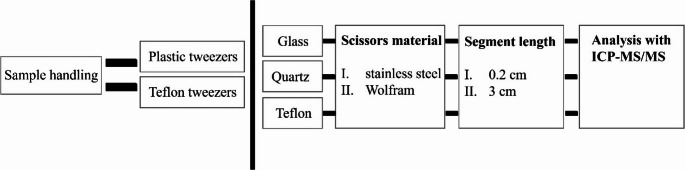
Schematic overview over the sample preparation development process. Except for the tweezer material testing with the purpose of evaluating the contamination potential, the sample preparation procedure involved all possible combinations of vial material, scissor material, and segment length. Real hair samples donated from a single volunteer were used for testing. The samples were processed within the same analytical batch to avoid instrumental bias

After evaluating sample handling materials, tweezers in particular, for contamination, three factors were considered for the development of a robust sample preparation method. Unlike liquid samples, such as urine and blood, hair provides a different set of challenges for the analyst that need to be aligned with the needs of HBM. Therefore, the method was developed according to the following workflow: (1) The achievable LODs and LOQs were evaluated for three types of digestion vessels using CRMs, (2) two types of scissors used to cut long hair samples to the required length were evaluated, and (3) to harmonize the need for a segment length achievable by the largest number of samples possible, while assuring homogeneity and allowing exposure assessment of the past three months, two segment lengths were considered and evaluated. For a complete overview, the method development is summarised in Fig. 1. Samples and CRMs were weighted on an XPE205 balance with a resolution of 0.01 mg. For the digestion, a standard method was used and equally applied to all evaluated sample preparation methods: 1 mL of 65% SupraPur HNO_3_ was used in a Milestone UltraWave single reaction chamber microwave digestion system (Italy) with a standard procedure (20 min temperature increase to 240 °C at 100 bar which is then kept for 15 min). After digestion, the samples were diluted to 10 mL with Milli-Q water.

#### Sample Pre-Treatment

All hair samples were cut near the scalp. Whether the samples should be pre-cleaned before analysis is an unresolved matter of debate. While it was recently demonstrated that pre-cleaning with Triton-ethanol or Triton-nitric acid has inconsistent effects on element concentrations in hair samples [19] other techniques have not been systematically evaluated. Thus, our study was designed in favour of comparability and follows the standard pre-cleaning procedure with acetone and Milli-Q water as recommended by the International Atomic Energy Agency [20].

Using test hair samples, we evaluated the suitability of two different sample handling materials (plastic and Teflon tweezers).

**Firstly**, the test hair samples were transferred to beakers and covered with acetone for 10 min. Afterwards, the samples were transferred to a vacuum filter using plastic tweezers. They were rinsed with Milli-Q water, followed by acetone, and rinsed again with Milli-Q water before being left to dry overnight in a clean room.

**Secondly**, the steps from the second test were repeated, but Teflon tweezers were used for sample handling instead of plastic tweezers.

#### Sample Treatment

##### Digestion Vials

For the digestion, we used vessels from three different materials: Teflon, glass, and quartz. Two CRMs, namely NIES No. 13 and IAEA-086, test hair samples, and three blanks were used to evaluate the suitability of the material in terms of (1) achievable LOD and LOQ, (2) element recoveries, and (3) reproducibility and robustness of the results. This was further evaluated for the two segment lengths described in Sect. 2.2.2.2 (3 cm and 0.2 cm).

##### Scissors

As standard stainless-steel hair scissors could potentially contaminate the test hair samples, Wolfram scissors (stainless-steel covered with tungsten carbide) were also tested to cut them to 3 cm and 0.2 cm, respectively. The results were compared for both scissors and sample lengths in three different digestion vessels: Teflon, glass, and quartz.

##### Hair segment length

As human scalp hair grows at an average rate of 0.35 mm per day [21] the choice of length of the sample segment is dependent on the exposure period of interest to the analyst and limited by the method detection limit, the homogeneity requirements, and the availability of hair samples at the required length. We excluded the possibility of analysing the entire hair length as this would cause incomparability among individuals with long hair, corresponding to several years of accumulated exposure, and short hair, corresponding to exposure periods as short as one month or less. To limit the necessary exclusion of individuals with shorter hair while providing enough samples to detect the analytes at levels above the LOD, it was decided to set the maximum length of a segment to 3 cm, which roughly captures the accumulated exposure over the past three months prior to sampling. With regard to sample homogeneity, digestion efficiency, and sample handling, we evaluated the digestion of the full segment (3 cm) and further cut it into smaller segments. The test hair sample was cut into 3 cm segments and divided into aliquots that were either kept whole or further cut into 0.2 cm segments before being transferred into the digestion vessel. The sample weight with either method was 20 mg. All sample handling materials were cleaned with ethanol between samples.

#### Instrumental Analysis

The instrumental analysis was performed on a Triple Quadrupole Inductively coupled plasma mass spectrometry (ICP-MS/MS) 8800, Agilent, Japan with the following isotopes included in the multi-elemental analytical method: ^107^Ag, ^27^Al, ^75^As, ^137^Ba, ^44^Ca, ^111^Cd, ^59^Co, ^52^Cr, ^63^Cu, ^56^Fe, ^201^Hg, ^39^K, ^24^Mg, ^55^Mn, ^98^Mo, ^23^Na, ^60^Ni, ^31^P, ^208^Pb, ^85^Rb, ^34^S, ^121^Sb, ^78^Se, ^118^Sn, ^88^Sr, ^47^Ti, ^238^U, ^51^V, and ^66^Zn. Fe was measured in H_2_ mode; P, Ti, Cr, As, and Se in O_2_ mode with mass shift (m_a_ + 16) and Ca without mass shift; and all other elements were measured in He mode. The instrument was equipped with a micro-mist nebuliser, a Scott-type spray chamber, and an ASX-510 (Cetac) autosampler. Y, Rh, Sc, and Gd were selected as internal standards based on their compatibility with the target analytes in terms of ionisation potential and mass-to-charge ratio, and their low abundance in environmental samples to prevent interferences. The internal standards were added online in all modes. A detailed overview of the instrumental settings is provided in supplementary Tables S1 and S2.

### Quality Control and Uncertainty

#### Contamination Control

The test hair samples were obtained from a single volunteer using Wolfram scissors. They were cut at the closest proximity to the scalp possible and transferred to a plastic storage bag, where they remained at room temperature until preparation. All sample treatment is carried out inside a clean room with a closed ventilation system to prevent contamination.

To control for contamination during the sample preparation, each digestion batch includes two CRMs (NIES No. 13 and IAEA-086) as well as two blanks. Both the CRMs and the blanks underwent complete sample preparation and analytical procedures.

#### Quality Control

To ensure the quality of the analytical method, two CRMs (NIES No. 13 Human Hair and IAEA-086 Human Hair, Btl. No. 1995/658) were included in each digestion batch. At least two blanks were included in each digestion. For the following elements, no CRMs were available: P, K, Ti, Cr, Ni, Rb, Sr, Mo, Sn, and U. The quality of the results was assessed via the RSDs of quadruple measurements and the uncertainties that were calculated for each element.

The first step of the method development was the determination of the LODs and LOQs for three types of digestion vials, namely glass, quartz, and Teflon. The LOD is determined as three times the standard deviation of blank samples divided by the slope of the calibration curve. To obtain the mass-corrected sample LOD, the LOD of the analysed solution is multiplied by the volume of the solution and divided by the average mass of the sample. The LOQs for each element were determined by multiplying the LOD with a factor of 3.3.

During the method development, the samples were prepared and digested in different batches but analysed in a single sequence with ICP-MS/MS to allow a comparison of sample preparation methods. While CRMs provide a reliable and homogeneous matrix for quality control, the composition of real hair samples might be heterogeneous, and the method’s performance might depend on the length of the hair segment. Therefore, a single volunteer provided a generous hair sample that was further divided into aliquots that were used for the method development. For each treatment, quadruples were prepared and analysed, and only elements with RSDs ≤ 20% in both CRMs and test hair samples were considered for the final method.

As a final aspect for consideration, the recoveries were calculated for those elements for which CRMs are available. The limit for recoveries was set to ± 20%.

#### Measurement Uncertainty

**Fig. 2:**
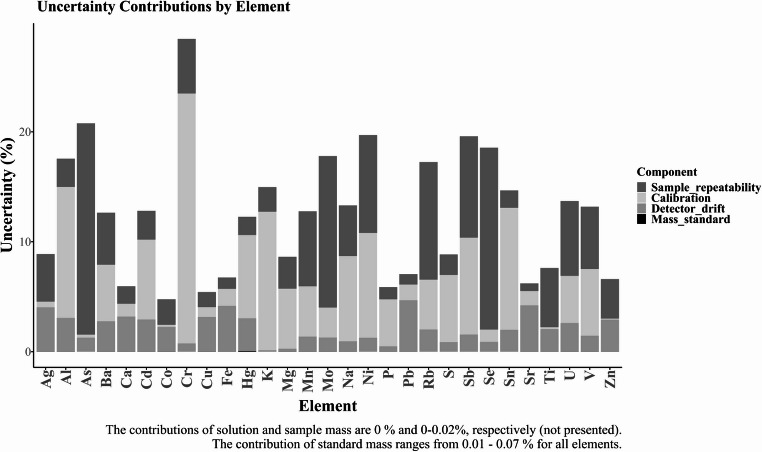
The expanded relative combined standard uncertainties (k = 2) for the determination of elements using ICP-MS/MS. The height of the stacked bar indicates U_ex_, while the contribution index of individual uncertainty source is represented by a respective colored part of each bar. The contributions of mass of the sample, solution, and standard were between 0% and 0.07% (Table S3)

The uncertainty of the measurement results was estimated using a modelling approach following the ISO-GUM/Eurachem guidelines [22, 23]. The concentrations of elements in hair samples determined by ICP-MS/MS were calculated using the corresponding mathematical model based on linear regression. Appropriate uncertainty sources (calibration and ICP drift, concentrations of certified standard solutions, repeatability, sample mass, and mass of digested solution) were quantified, converted to standard uncertainties, and pooled together following the rules for uncertainty propagation. The expanded relative combined standard uncertainties were expressed using a coverage factor of k = 2. The expanded relative combined standard uncertainties (k = 2) are illustrated in Fig. 2, and the individual contributions of sample repeatability, calibration curve, the masses of the solution, sample, and standard, and the detector’s drift to the combined relative uncertainty are presented in supplementary Table S3. The calculated expanded uncertainties (k = 2) were as follows: Ag 8.9%, Al 17.6%, As 20.8%, Ba 12.7%, Ca 6.0%, Cd 12.8%, Co 4.8%, Cr 28.5%, Cu 5.4%, Fe 6.8%, Hg 12.3%, K 15.0%, Mg 8.6%, Mn 12.8%, Mo 17.8%, Na 13.3%, Ni 19.7%, P 5.9% Pb 7.1%, Rb 17.3%, S 8.9%, Sb 19.6%, Se 18.6%, Sn 14.7%, Sr 6.2%, Ti 7.6%, U 13.7%, V 13.2%, and Zn 6.6%.

#### Method Application

The optimised and validated method is currently being applied in a national HBM study on 1335 children and adolescents from Slovenia sampled between 2020 and 2024 (HBM II). The preliminary results are presented in this manuscript. The study protocol was approved by the National Medical Ethics Committee of the Republic of Slovenia (number of accordance: 0120–431/2018/7). Informed written consent was obtained from the legal guardians of all participants.

#### Visualisation of the Results

All figures were created in R Version 4.3.3 [24] and RStudio Version 2024.12.1.563 [25] using the package ggplot2 [26].

## Results

### Sample Pretreatment

To reduce the probability of contamination, the sampling handling materials should be cautiously selected and tested. Therefore, we chose plastic and Teflon tweezers for direct contact with the samples. We did not observe contamination from either of the materials, and cleaning the tweezers with ethanol between each sample helped to reduce static electricity in the hair samples.

### Sample Preparation

**Fig. 3:**
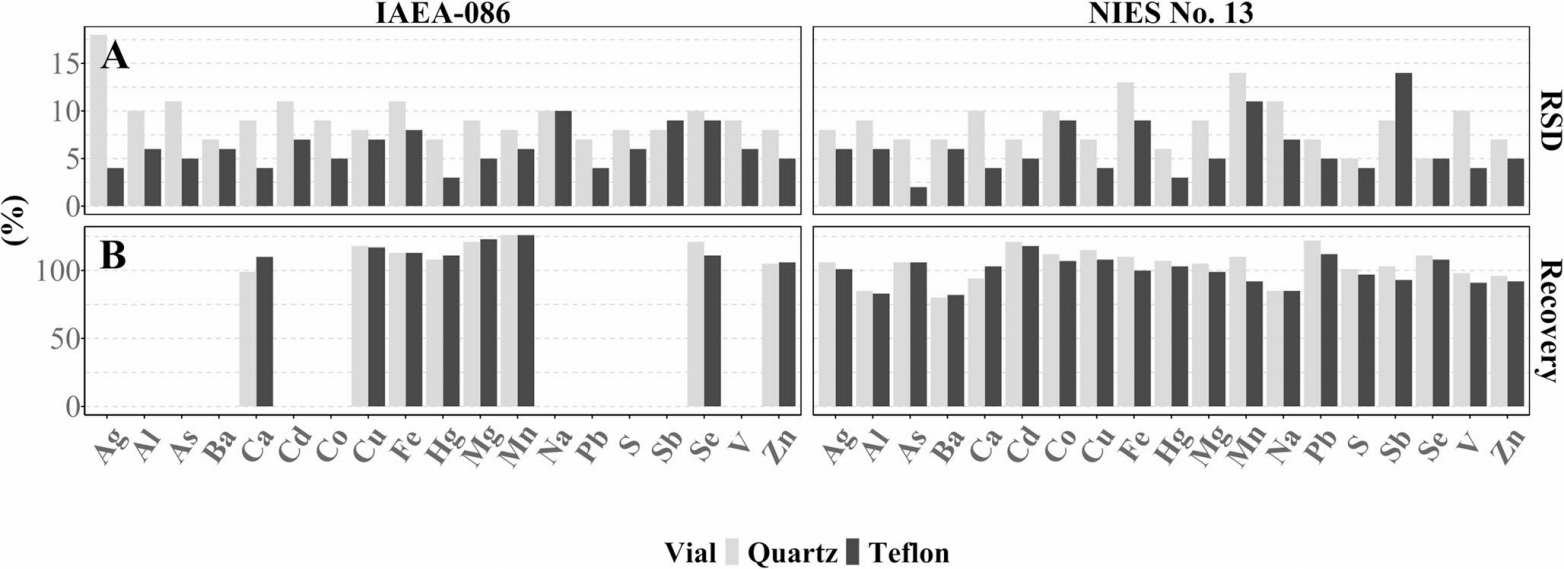
(**A**) RSDs (%) and (**B**) recoveries obtained for CRMs IAEA-086 and NIES No. 13 in Teflon and quartz vials

**Fig. 4:**
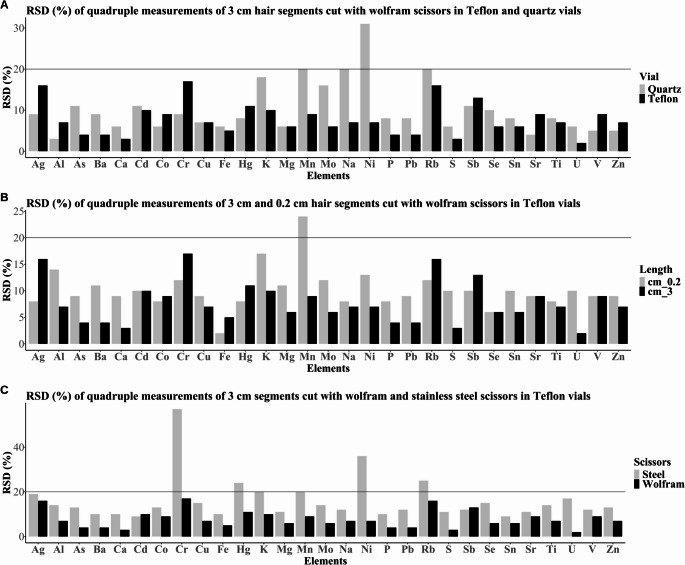
RSD (%) obtained for different sample preparation methods. (**A**) RSD (%) of Teflon and quartz vials, (**B**) RSD (%) of measurements in 3 cm and 0.2 cm hair segments, and (**C**) RSD (%) of 3 cm hair segments cut with Wolfram and stainless-steel scissors

The results of the method development procedure of sample preparation are summarised in Figs. 3 and 4; Table 1 as well as Table S5. Due to its extent, a table summarising the mean concentrations and relative standard deviations of quadruplets analysed for each sample treatment procedure has been included in the supplementary material (Table S4).

**Table 1:** Presented are the LODs and LOQs achieved for glass, quartz, and teflon vessels (ng/g). For comparison, we included the certified reference values (ng/g) and the unpublished results from an ongoing National HBM study, including 1335 children and adolescents, to which the method is currently being applied

LOD (LOQ) in ng/g				CRM reference values		HBM II	% < LOD		% < LOQ	
Element	glass	quartz	Teflon	IAEA 086		GM (ng/g)^a^	quartz	Teflon	quartz	Teflon
Ag	5 (16.5)	5 (16.5)	10 (33)		100	14.45	16.3	35.0	51.3	74.8
Al	1.15 × 10^5 (3.79 × 10^5)	790 (2607)	470 (1551)		1.2 × 10^5	5152.76	4.3	2.7	17.8	9.4
As	3 (9.9)	2 (6.6)	1 (3.3)		100	16.96	0.1	0.1	1.3	0.2
Ba	240 (792)	20 (66)	10 (33)		2000	274.78	0.3	0.1	4.1	0.6
Ca	2.3 × 10^4 (7.59 × 10^4)	1.5 × 10^4 (4.95 × 10^4)	3.2 × 10^4 (1.06 × 10^5)	1.12 × 10^6	8.2 × 10^5	3.778 × 10^5	0.0	0.1	0.1	0.8
Cd	0.9 (2.97)	0.4 (1.32)	1 (3.3)		230	11.89	0.0	0.3	0.5	6.9
Co	4 (13.2)	1 (3.3)	2 (6.6)		70	17.32	0.4	2.5	7.5	24.6
Cr*	300 (990)	30 (99)	75 (247.5)			74.75	17.1	51.5	62.5	85.6
Cu	140 (462)	240 (792)	70 (231)	1.76 × 10^4	1.53 × 10^4	1.133 × 10^4	0.0	0.0	0.0	0.0
Fe	6000 (19800)	1000 (3300)	700 (2310)	1.23 × 10^5	1.4 × 10^5	6779.02	0.1	0.1	0.4	0.1
Hg	10 (33)	10 (33)	10 (33)	573	4420	164.53	0.1	0.1	4.7	4.7
K*	1.83 × 10^5 (6.04 × 10^5)	2000 (6600)	6000 (1.98 × 10^4)			8.21 × 10^4	2.0	8.2	8.9	23.9
Mg	6000 (19800)	650 (2145)	400 (1320)	1.77 × 10^5	1.6 × 10^5	3.483 × 10^4	0.0	0.0	0.0	0.0
Mn	80 (264)	20 (66)	35 (115.5)	9600	3900	117.43	1.4	5.3	21.9	49.9
Mo*	60 (198)	10 (33)	5 (16.5)			32	0.7	0.3	56.8	4.9
Na	8.6 × 10^5 (2.84 × 10^6)	8800 (2.90 × 10^4)	8.2 × 10^3 (2.71 × 10^4)		6.1 × 10^4	1.515 × 10^5	2.0	2.1	12.1	12.3
Ni*	130 (429)	30 (99)	60 (198)			73.87	12.4	19.9	37.9	72.4
P*	240 (792)	280 (924)	300 (990)			1.23 × 10^5	0.0	0.0	0.0	0.0
Pb	20 (66)	4 (13.2)	4 (13.2)		4600	342.41	0.0	0.0	0.1	0.1
Rb*	120 (396)	3 (9.9)	2 (6.6)			81.15	2.0	0.9	12.2	7.4
S	1.5 × 10^5 (4.95 × 10^5)	8 × 10^4 (2.64 × 10^5)	1.05 × 10^5 (3.47 × 10^5)		5 × 10^7	4.66 × 10^7	0.0	0.0	0.0	0.0
Sb	1 (3.3)	0.8 (2.64)	0.9 (2.97)		42	14.06	0.1	0.2	2.4	3.5
Se	7 (23.1)	10 (33)	15 (49.5)	1000	1790	453.73	0.0	0.0	0.0	0.1
Sn*	9 (29.7)	10 (33)	7 (23.1)			61.58	1.3	0.4	24.6	11.7
Sr*	120 (396)	60 (198)	15 (49.5)			445.99	0.9	0.2	22.8	0.5
Ti*	200 (660)	300 (990)	100 (330)			911	7.4	0.4	53.0	9.2
U*	2 (6.6)	0.2 (0.66)	0.3 (0.99)			16.65	0.0	0.0	0.1	0.6
V	10 (33)	1 (3.3)	0.9 (2.97)		270	12	2.2	2.1	5.5	4.6
Zn	3000 (9900)	4400 (1.45 × 10^4)	4000 (1.32 × 10^4)	1.67 × 10^5	1.72 × 10^5	1.69 × 10^5	0.0	0.0	0.0	0.0

*no reference value is available for this element; ^a^Samples were digested in quartz vials.

#### Sample Digestion Vials

The choice of sample digestion vessel had the largest impact on the overall method performance. Three different digestion vessels were tested: glass, Teflon, and quartz. The most suitable vessel was chosen based on (1) the obtained LOD and LOQ, (2) the relative standard deviation (RSD) of mean concentrations in CRMs and test hair samples, and (3) the overall recovery (%).

The limit of detection is the most important criterion based on which the digestion vessel was chosen (Table 1). As the numbers indicate, the LODs achieved in glass are unacceptably high for Ba, Cr, Mn, Mo, Ni, Rb, and V in comparison with the geometric means obtained from an ongoing national HBM campaign of 1335 children and adolescents (HBM II; unpublished results). The LOQs for 12 elements lie above the geometric mean (Table 1). In Teflon, the LODs obtained for Cr and Ni, and the LOQs for Ag, Cr, Mn, and Ni are either above or close to the geometric mean in HBM II. If digested in quartz vials all obtained LODs lie below the HBM II geometric means. Only for the LOQs for Mo and Ti does Teflon offer benefits over quartz. When comparing the detection frequencies in HBM II, the highest percentage of samples below the LOD was obtained for Ag (16%), whereas 51%, 63%, 57%, and 53% of samples were below the LOQs for Ag, Cr, Mo, and Ti. Considering the LOQs obtained for those elements after digestion in Teflon vials, the numbers increase to 75% and 86% for Ag and Cr and decrease to 5% and 9% for Mo and Ti. However, 50% and 72% of samples are below the LOQ for Mn and Ni after digestion in Teflon (Table 1). Thus, we believe that both Teflon and quartz vials generally yield appropriate LODs and LOQs for HBM with quarts being slightly superior in terms of the number of detectable elements.

Figure 3A presents the RSDs (%) for Teflon and quartz vials for two CRMs. The exact values for Teflon, quartz, and glass vials are presented in supplementary Table S6. The RSD (%) obtained in glass vials exceeded the 20% limit for Al, Mn, and Na (Table S6). This is a further indication to exclude glass from any further method development. In Teflon and quartz, the RSDs were comparable and satisfactory for all elements, though generally lower in Teflon than in quartz vials (Table S6). In 3 cm test hair segments cut with Wolfram scissors (Fig. 4A, Table S4), the results based on the RSD of the mean concentrations were acceptable (< 20% RSD) for most elements; however, Ni exceeded the limit in quartz vials (RSD 31%), which could potentially be lowered by increasing the sample mass However, this might be challenging to acquire from participants with short hair. The RSDs for elements after digestion in Teflon vials ranged from 3% to 17%. However, after digestion in glass vials, the RSDs for eight elements (Al, As, Cr, K, Mo, Na, Ni, and Rb) exceeded 20% (Table S4). The results of the test hair samples and the CRMs allow the conclusion that quartz and Teflon are suitable for the digestion of hair samples for multi-elemental analysis. The recovery of elements was evaluated using the CRMs IAEA-086 and NIES No. 13. As presented in Fig. 3B; Table 2, and Table S6, after digestion in Teflon the recoveries for Mg (123%) and Mn (126%) exceed the upper limit of ± 20% in CRM IAEA-086. After digestion in glass vials, the recovery of Cd (126%), Na (824%), and Pb (123%) exceeded the limits for NIES No. 13. If digested in quartz vials, the upper limit was exceeded for Mg (121%), Mn (126%, and Se (121%) for CRM IAEA-086 and for Cd (121%) and Pb (122%) for CRM NIES No. 13 (Table S6). When the average recovery obtained from both CRMs is calculated, only Cd (121%) and Pb (122%) exceed the limit after digestion in quartz vials.

**Table 2:** Summary of the analytical method parameters. The accuracy is the average accuracy obtained for both CRMs, except for those elements included in only one CRM

Element	LOD (ng/g)	LOQ (ng/g)	Recovery (%)	RSD (%)	Linearity (*r*^2^)	Uncertainty (%)
Ag	5	16.5	106	13	0.999	8.9%
Al	790	2607	85	10		17.6%
As	2	6.6	106	9		20.8%
Ba	20	66	80	7		12.7%
Ca^a^	1.5 × 10^4	4.95 × 10^4	96	10		6.0%
Cd	0.4	1.32	121	9		12.8%
Co	1	3.3	112	9		4.8%
Cr^b^	30	99		9		28.5%
Cu^a^	240	792	117	8		5.4%
Fe^a^	1000	3300	112	12		6.8%
Hg^a^	10	33	108	7		12.3%
K^b^	2000	6600		18		15.0%
Mg^a^	650	2145	113	9		8.6%
Mn^a^	20	66	118	11		12.8%
Mo^b^	10	33		16		17.8%
Na	8800	2.90 × 10^4	85	11		13.3%
Ni^b^	30	99		31		19.7%
P^b^	280	924		8		5.9%
Pb	4	13.2	122	7		7.1%
Rb^b^	3	9.9		20		17.3%
S	8 × 10^4	2.64 × 10^5	101	6		8.9%
Sb	0.8	2.64	103	9		19.6%
Se^a^	10	33	116	8		18.6%
Sn^b^	10	33		8		14.7%
Sr^b^	60	198		4		6.2%
Ti^b^	300	990		8		7.6%
U^b^	0.2	0.66		6		13.7%
V	1	3.3	98	10		13.2%
Zn^a^	4400	1.45 × 10^4	101	8		6.6%

^a^ Element included in both CRMs; ^b^ no reference value is available for this element. RSD (%) were obtained from test hair segments

#### Scissors

Standard hair scissors are commonly made from stainless steel, primarily composed of iron and carbon and additional alloys, such as Ni and Mn [13, 27]. Alternatively, Wolfram scissors may be used if the element is not included in the measurement. We tested the effect of scissors on test hair samples.

**Fig. 5:**
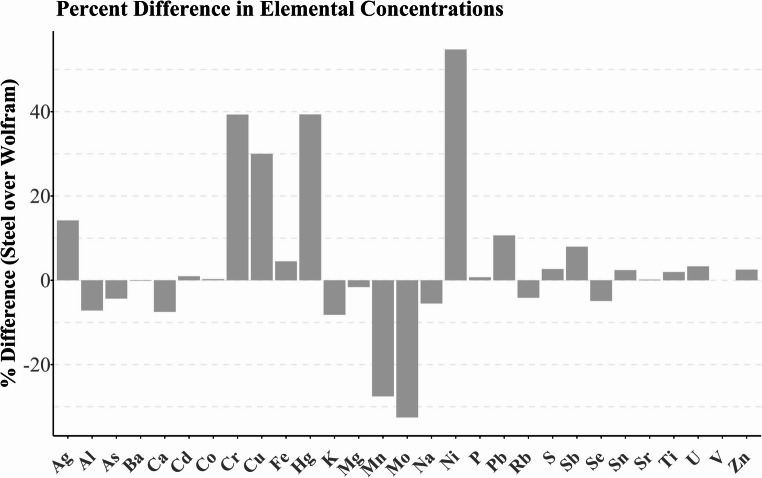
Percentage difference in elemental concentrations in 3 cm test hair segments cut with stainless steel scissors compared to segments cut with wolfram scissors

As expected, the choice of material strongly affected the results. The concentrations of elements were on average 4% higher when the samples were cut with stainless-steel scissors (Fig. 5). The difference is especially evident for Cr, Cu, Hg, and Ni, where the average concentration was increased by 39%, 30%, 39%, and 55% when stainless-steel scissors were used. However, the concentrations of Mn and Mo were on average 28% and 33% lower. As presented in Fig. 4C, the RSDs were significantly worse for samples cut with stainless-steel scissors, which leads to increases in uncertainty when these scissors are used. Therefore, we recommend using Wolfram scissors to cut the hair samples into aliquots of the appropriate length to avoid contamination.

#### Sample Segment Length

It is widely accepted that the inhomogeneity of hair samples is a source of uncertainty in hair analysis-based HBM. To the best of our knowledge, no studies exist to date that would evaluate the effect of sample segment length on the measurement results. Therefore, we considered this.

by comparing the measurement results and method performance for two segment lengths cut with Wolfram scissors.

At different segment lengths (0.2 cm and 3 cm), but equal weight (20 mg), most elements performed well, meaning that the difference between the mean concentrations at both lengths was within ± 20% (Fig. 4B). Only Mn exceeded the 20% limit in 0.2 cm segments. Though both seem appropriate, the RSDs are lower in 3 cm segments compared to 0.2 cm segments. As we expect higher uncertainties at concentrations around the LOD, which could occur during HBM studies, we prefer the more robust results obtained for 3 cm segments.

### Instrumental Analysis

The performance of the instrumental analysis was monitored through the inclusion of CRMs, the linearity of the calibration curve, and the stability of the internal standard. The method proved to be robust, reproducible, and sensitive throughout the measurements, with a linearity r^2^ > 0.999 and good recoveries of most of the elements included in two CRMs. It is continuously applied in national biomonitoring studies in Slovenia.

**Therefore**,** the final method is as follows**:

Hair samples are cut to 3 cm segments using Wolfram scissors and left fully covered in acetone for 10 min. Afterwards, they are transferred to a vacuum filter using plastic tweezers. After additional rinsing with Milli-Q, acetone, and again with Milli-Q, the samples are covered and left to dry overnight in a clean room.

20 mg of the sample is transferred into a quartz digestion vessel with plastic tweezers. Each digestion batch contains the CRMs NIES No. 13 and IAEA-086, as well as two blanks, ten samples, and one parallel. The samples are digested in quartz vessels with 1 ml of 65% SupraPur HNO_3_ with the following digestion settings: A 20-minute temperature increase to 240 °C and 100 bar, which is to be kept for 15 min. After digestion, the samples are diluted to 10 mL with fresh Milli-Q water. The analytical method parameters are summarised in Table 2. The method is currently being applied in an ongoing national HBM campaign, and the geometric means and detection frequencies are presented in Table 1.

### Overview of Published Methods

An overview of published methods is presented in Table 3 (*N* = 19). The most frequently determined elements were Pb (*N* = 18), Cu (*N* = 17), Zn (*N* = 16), Cd (*N* = 15), and Cr (*N* = 14). As, Fe, and Mn were each included in eleven methods, Co and Ni in ten methods, and Se in nine methods. Moderately frequently included were Hg and Li (*N* = 7), Al, Ca, and Mg (*N* = 6), Ba, Be, K, Si, Sn, and V (*N* = 5), Ag, Bi, Mo, P, Sb, and Sr (*N* = 4), B, Na, and Tl (*N* = 3), and Ce, I, Rb, U, and W (*N* = 2). Au, Br, Cs, Eu, Ga, Gd, Hf, In, La, Nb, Nd, Pt, Re, S, Sc, Th, Ti, Y, Yb, and Zr were each included in only one method.

**Table 3:** An overview of methods for multi-elemental analysis of human scalp hair samples

		Sample pre-treatment		Sample treatment			Analysis		Reference
Segment length	Sample weight (g)	Sampling tool	Pre-cleaning method	Digestion solution	Digestion system	Digestion vessel	Instrument	Analytes	
3 cm	0.2	Wolfram scissors	Acetone, Milli-Q water	HNO_3_	Microwave digestion	Quartz vessel	ICP-MS/MS	Ag, Al, As, Ba, Ca, Cd, Co, Cr, Cu, Fe, Hg, K, Mg, Mn, Mo, Na, Ni, P, Pb, Rb, S, Sb, Se, Sn, Sr, Ti, U, V, Zn	This study
≤ 2.5 cm	0.6–0.8	Stainless-steel scissors	no washing	HNO_3_	25 min incubation	Centrifuge tube	ICP-MS	B, Ca, Cr, Co, Cu, Fe, Mg, Mn, Mo, Ni, P, K, Se, Si, Na, Zn, Al, Sb, Ba, Be, Bi, Cd, Pb, Li, Hg, Pt, Tl, Sn	[14]
Full length	0.5	NA	Acetone, ultrapure water	65% HNO_3_	Microwave digestion	NA	ICP-MS	Pb, Cd, As, Zn, Cu, Fe, Se	[28]
Full length	0.1	Ceramic scissors, planetary ball mill	Acetone, deionised water	TMAH	Microwave digestion	NA	ICP-MS	S, Si, Ca, Br, Fe, Cu, Cr, Mg, Si, K, Mn, Ni, Zn, Se, Sr, Pb	[29]
1–2 cm	0.15	Stainless-steel scissors	Acetone, deionised water	HNO_3_, H_2_O_2_	48 h Incubation	Glass beaker	ICP-MS	Al, As, Ba, Cd, Co, Cr, Cu, Fe, Li, Mn, Mo, Ni, Pb, Rb, Sb, Se, Sr, U, V, Zn	[30]
Full length	0.2	Stainless-steel scissors	Acetone, milli-Q water	HNO_3_, H_2_O_2_	Microwave digestion	NA	ICP-MS	As, Pb, Cd, Mn, Cu, Co, Fe, Zn, Se	[31]
Full length	1	NA	Acetone, detergents, distilled water	69% HNO_3_, 70% HClO_4_, 98% H_2_SO_4_	Incubation on a hot plate	NA	AAS	Cd, Cu, Pb, Zn	[32]
Full length	0.1	NA	Acetone, ultrapure water	H_2_O_2_, HNO_3_, HClO_4_	Over-night incubation	Teflon	ICP-MS	Cu, Zn, Pb, Cd, Se, Cr, As, Hg	[33]
Full length	0.1	Ceramic scissors	Acetone, ultrapure water	69% HNO_3_, 30% H_2_O_2_	Microwave digestion	NA	ICP-MS	As, Cd, Cr, Sb, Pb, Hg	[34]
Full length	0.2	Stainless-steel scissors	2% neutral detergent, ultrapure water	2:1 HNO_3_/H_2_O_2_	NA	NA	ICP-MS	Cd, Cr, Cu, Mn, Ni, Pb, Zn	[35]
1 cm	0.25	Stainless-steel scissors	Acetone, ultrapure water, 1% Triton X-100	HNO_3_	Microwave digestion	Easy Prep Plus^®^ vessels (Teflon)	ETAAS	Ag, Bi, Cd, Pb	[36]
Full length	0.15	Ceramic scissors	Acetone, deionised water	70% HNO_3_, 30% H_2_O_2_	10 min incubation in a hot water bath	NA	SN-ICP-MS	Li, Mg, Cr, Mn, Fe, Co, Ni, Cu, Zn, Sr, Ag, Ba, Hg	[37]
Full length	0.2	NA	2% Triton X-100, distilled water	65% HNO_3_	Over-night incubation	Polypropylene tube	ICP-MS	Al, Ag, As, Au, B, Ba, Be, Bi, Ca, Cd, Ce, Co, Cr, Cu, Fe, Ga, Hg, K, Li, Mg, Mn, Mo, Na, Ni, Pb, Rb, Re, Sb, Se, Sn, Sr, Ti, Tl, V, W, Zn	[38]
Full length	2	Stainless-steel scissors	Detergent, deionised water	65% HNO_3_, 30% H_2_O_2_	Hotplate incubation	Teflon	ICP-MS	As, Cd, Cr, Cu, Pb, Zn	[39]
Full length	1	Non-metallic scissors	no washing	30% H_2_O_2_, 65% HNO_3_	Microwave digestion	Teflon	ICP-MS	Cd, Cr, As, Co, Ni, Pb, Cu	[40]
Full length	0.1–0.5	NA	non-ionic detergent, acetone, deionised water	69% HNO_3_	Microwave digestion	Teflon	ICP-AES	Cu, Zn, Co, Fe, Cr, Mn, Cd, As, Pb, Ni, P, Ca, K and Mg	[16]
3 cm	0.2	Steel grinder jar with steel beads	0.1% Triton X 100 solution, acetone, deionised water	69% HNO_3_, 30% H_2_O_2_	Microwave digestion	TFM (modified PTFE)	ICP-AES	Al, Pb, Mg, K, P, Ca, Zn, Cu, Fe	[18]
Full length	0.2–0.3	Stainless-steel scissors	acetone, deionised water	HNO_3_	Microwave digestion	Teflon	ICP-MS	Ag, Ba, Be, Bi, Cs, Co, Ce, Cr, Cu, Eu, Gd, Hf, In, La, Li, Mn, Mo, Nb, Nd, Pb, Sc, Sn, Tl, Th, U, V, W, Y, Yb, Zn, Zr	[11]
0.5–1 cm	0.1	NA	acetone, deionised water	HNO_3_	NA	Teflon	ICP-MS	Al, As, Be, Cd, Co, Cr, Cu, Fe, Hg, I, Li, Mn, Ni, Pb, Se, Si, Sn, V, Zn	[10]
Full length	0.05–0.1	Stainless-steel scissors	acetone, deionised water	HNO_3_	Microwave digestion	Teflon	ICP-DRC-MS	Ca, K, Mg, Na, P, Co, Cr, Cu, Fe, I, Li, Mn, Se, Si, V, Zn, Al, As, B, Be, Cd, Hg, Ni, Pb, Sn	[41]

*NA* information not available.

The majority of studies (*N* = 14) digest the full length of the sample [11, 16, 28, 29, 31–35, 37–41], four studies digest 1 to 3 cm segment lengths [14, 18, 30, 36], and one method utilises segment lengths of less than 1 cm [10]. The most commonly used sample weight is between 0.1 g and 0.5 g [10, 11, 16, 18, 29–34, 38, 39, 42], with four methods using higher weights [14, 32, 39, 40], and one method using less than 0.1 g of sample [41].

The most commonly described washing procedure is a combination of water and acetone (*N* = 10) [10, 11, 28–31, 33, 34, 37, 41], while different combinations of acetone, water, detergent, and Triton X-100 are described as well [16, 18, 32, 35, 36, 38]. Two methods describe the digestion and analysis of unwashed samples [14, 40].

Eight studies report cutting the hair samples with stainless-steel scissors [11, 14, 30, 31, 35, 36, 39, 41] and one describes the use of a steel grinder jar with steel beads [18]. Three methods utilise ceramic scissors [29, 34, 37], and one study describes the material as non-metallic [40]. Six methods do not disclose the material [10, 16, 28, 32, 33, 38].

One study describes the digestion in glass beakers [30], one digests the samples in polypropylene tubes [38], and a third study reports the use of centrifuge tubes [14]. The most used material is Teflon, as reported by nine studies [10, 11, 16, 18, 33, 36, 39–41]. Seven studies do not describe the material of the digestion vials [28, 29, 31, 32, 34, 35, 37].

## Discussion

### Method Limitations

While this study provides a solid basis for method development, a few limitations should be noted that result of a compromise between analytical precision and practicalities in HBM Firstly, increasing the sample mass would likely increase repeatability, reproducibility and accuracy of the results, specifically for Pb and Cd. However, in the pilot study of 1335 children and adolescents, acquiring even a sample mass of 20 mg without visible damage to the hair cut can be challenging from participants with short hair. The consequences are either missing samples from those participants or reduced sample mass, the former reducing statistical power in further data analyses and the latter disproportionally increasing the uncertainty for a subset of the samples. Secondly, hair segments of 3 cm can generally not be obtained from all participants of a human biomonitoring study. However, in favour of results reproducibility and LOD for some of the elements, 3 cm segments are preferred. This challenge can be circumvented by including more sample replicates or by excluding individuals with hair shorter than 3 cm if the study design allows it. Furthermore, hair growth rate and structure can vary between individuals, potentially affecting how elements are incorporated, which may limit broader applicability. The method focuses on total element concentrations without speciation, which is particularly relevant for elements like arsenic and mercury, where toxicity depends on chemical form. Finally, although it’s common practice in method validation, using only one hair donor does not account for inter-individual variability and should be considered in future work.

### Advantages of the Presented Method Over Conventional Methods

Despite its feasibility, hair analysis is underrepresented in HBM studies. However, it can yield information on long-term exposure that would be neglected if the interpretation of the results relied solely on urine and blood analysis. The approach of hair analysis in HBM is, to date, not unified; however, some overall guidelines are commonly accepted and applied, such as the standardised hair-washing procedure suggested by the International Atomic Energy Agency (IAEA) [43]. While no systematic studies exist on the variation of elemental composition in different hair segments, samples are most commonly collected at the occipital region close to the scalp [15]. Pozebon et al. (2017) present different materials used for sample acquisition, convincingly arguing that stainless-steel should be avoided due to proven contamination. They recommend the use of other materials, such as tantalum. Despite this, stainless-steel remains the preferred material in numerous studies (Table 3) analysing, among other elements, Cr, Cu, Hg, and Ni, for which we observed unsatisfactory results after contact with stainless-steel. Our results confirm the risk of contamination arising from using stainless-steel scissors and the benefits of Wolfram scissors used in the present study.

The effect of sample washing is thoroughly discussed by Pozebon et al. (2017) and David et al. (2023). They argue that while removing exogenous contamination is a crucial step in hair analysis, strong solvents, such as methanol, can also remove endogenous compounds. The individual losses differ among the analytes depending on differences in interaction and attachment. Unfortunately, no washing procedure removes solely exogenous compounds [13]. The method proposed by the IAEA in the 1970 s consists of a 5–10-minute washing procedure with acetone and deionised water and is still the most applied procedure [43]. Few studies report an alternative approach, including additional steps using a non-ionic detergent [16] and a 0.1% Triton X-100 solution [18]. Unfortunately, CRMs of unwashed human hair do not exist, so the effect of washing cannot be reliably evaluated [13]. Without thoroughly evaluated procedures and in favour of method comparability, we applied the IAEA method in this study.

As demonstrated in Table 3, only some studies provide details on the material of the digestion vessels, which indicates that its impact on the measurement results is not a widely considered aspect. Where details were provided, Teflon vessels were mostly used as they are the standard digestion vessels in microwave oven digestion, though their suitability is not evaluated by the studies; neither is this aspect mentioned in the review articles by Pozebon et al. (2017) [13] and Lum et al. (2021) [15]. In the present study, we demonstrated the importance of the digestion vessel material. Glass seems unsuitable for about a third of the elements included in this study.

One method presented in Table 3 describes the digestion of hair samples in glass beakers, before analysing 20 elements. Among those elements, our results suggest that glass might be unsuitable for 8, namely Al, As, Mo, Ni, Rb, Cr, and Mn; however, reproducible detection was possible when the samples were digested in Teflon and quartz vessels. Therefore, within this study, we would like to highlight the importance of carefully selecting the digestion vessel material, which is a neglected aspect in multi-elemental hair analysis.

Among the presented studies, microwave digestion is a common practice as it significantly shortens the digestion time, reduces the loss of compounds through closed vessels, and increases digestion efficiency [13]. However, different procedures are available regarding the choice of digestion solution, and Liu et al. (2022) demonstrated that programmed microwave digestion, microwave oven digestion, and digestion at room temperature are suitable methods. ICP-MS can be sensitive to stronger acids that can cause large interferences in the system. For instance, analysing halogens can be challenging, and they might form volatile species, leading to analyte loss. Due to these considerations, concentrated HNO_3_ is the most commonly used acid in hair digestion, often in combination with H_2_O_2_. If halogens are included in the method, tetramethylammonium hydroxide (TMAH) is a widely accepted alternative, though one study reported its use in a multi-elemental analysis, highlighting its wider suitability [29]. HNO_3_, despite being overall suitable for hair digestion, has been associated with unacceptably low signals for Hg [13]. However, we did not observe this in the present study.

To conclude, the main differences between the presented and numerous published methods lie in the careful evaluation of the material used for sample cutting and the digestion vessel material. Through this study, it becomes evident that the quality of analysis results can depend on these steps, and none of the methods published in recent years elaborated on the matter. Therefore, the present study adds valuable information to the existing pool of sample preparation methods for hair analysis in HBM.

## Conclusions

In the present study, we developed a sensitive and robust analytical method for the determination of Ag, Al, As, Ba, Ca, Cd, Co, Cr, Cu, Fe, Hg, K, Mg, Mn, Mo, Na, Ni, P, Pb, S, Sb, Se, Sn, Sr, Ti, U, V, and Zn in 3-cm segment of human hair.

Human hair is an important matrix in human biomonitoring as it complements the information obtained from blood and urine samples. Additionally, it may facilitate the sampling of small children who might hesitate to give a blood sample. Therefore, there is a need for robust and sensitive methods for the multi-elemental analysis of hair samples.

The presented method involves a washing procedure with acetone and Milli-Q water to effectively clean the hair samples from external contamination. 20 mg of 3 cm hair segments are digested in an UltraWave microwave digestion system in quartz vessels with 65% SupraPur HNO_3_ and diluted to a final volume of 10 mL before analysis with ICP-MS/MS.

Important results from the method development are as follows: Wolfram hair scissors are preferred over standard stainless-steel hair scissors to avoid contamination. Most elements had higher standard deviations among sample replicates, worse recoveries, and higher detection limits if digested in glass vials. Therefore, these should be avoided in favour of quartz or Teflon vessels. The presented method is now regularly applied in national HBM campaigns.

## Supplementary Information

Below is the link to the electronic supplementary material.

ESM 1 (DOCX 55.7 KB)

## Data Availability

The individual results of the national human biomonitoring study cannot be shared openly to protect the study participants’ privacy. Data are located in controlled access data storage at the Department of Environmental Sciences of the Jožef Stefan Institute. All data supporting the findings of the method development process are available within the paper and its Supplementary Information.
