# Supplementary material for: Optimisation of A Sample Preparation Method for the Determination of Multi-Elemental Compositions in Human Hair By Triple Quadrupole ICP-MS Analysis
Source: Biol Trace Elem Res. 2026 Jan 20;204(6):4348–62. doi: 10.1007/s12011-025-04968-5 (PMC13157397; doi:10.1007/s12011-025-04968-5)
Supplement: Supplementary file 1 — (DOCX 55.7 KB) [file 12011_2025_4968_MOESM1_ESM.docx]

Optimisation of a sample preparation method for the determination of multi-elemental compositions in human hair by triple quadrupole ICP-MS analysis

Agneta A. Runkel^1,3*^, Marta Jagodic Hudobivnik^1^, Igor Živković^1,2^, Polona Klemenčič^1^, Darja Mazej^1^, Milena Horvat^1,2*^

1. Jožef Stefan Institute, Jamova cesta 39, 1000 Ljubljana, Slovenia

2. ’Jožef Stefan’ International Postgraduate School, Jamova cesta 39, 1000 Ljubljana, Slovenia

3. Lund University, Division of Occupational and Environmental Medicine, Scheelevägen 8, 22363 Lund, Sweden

*corresponding author: agneta.runkel@ijs.si

**Supplementary material**

Table S1: Measured isotopes: integration times and stabilization times across tune modes

| Tune mode | #1: He | #2: H2 | 3#: O2 |
| --- | --- | --- | --- |
| stabilization time (s) | 30 | 15 | 30 |
| IntegTime/Mass (s) for isotope of Na, Mg, P, S, K Ca, Ti, Sr, Rb, Sr, Mo, Ag, Sn, Sb, Ba and Tl | 0.1000 | | |
| IntegTime/Mass (s) for isotope of Al, Sc, V, Cr, Mn, Fe, Co, Ni, Cu, Y, Rh, Gd, Pb and U | 0.3000 | | |
| IntegTime/Mass (s) for isotope of As, Se, Cd and Hg | 1 | | |

Table S2: ICP‑MS instrumental operating conditions

| Tune mode | **He** | | **H_2_** | | **O_2_** |
| --- | --- | --- | --- | --- | --- |
| Radio Frequency Power (RF Power) | 1550 W | | | | |
| Plasma gas flow (cooling gas, Ar) | 15 L/min | | | | |
| Carrier gas flow | 0.95 L/min | | | | |
| Makeup gas flow | 0.1 L/min | | | | |
| CRC gases flow | 3.0 mL/min | | 7.0 mL/min | | 30% |
| kinetic energy discrimination voltage | 5.0 V | | 0.0 V | | -7.0 V |
| Sampling Depth (mm) | 8.0 | | | | |
| RF Matching (V) | 1.6 | | | | |
| Nebulizer Pump (rps) | 0.1 | | | | |
| Spray chamber temperature (S/C Temp) | 2 °C | | | | |
| Octopole RF voltage (V) | 130 | 150 | | 200 | |
| Lens voltages: Extract 1 (V) | 0 | | | | |
| Extract 2 (V) | -200 | | | | |
| Omega Bias (V) | -105 | -95 | | -110 | |
| Omega Lens (V) | 9.1 | 9.1 | | 8.7 | |
| Q1 Entrance (V) | -3.0 | -4.0 | | -9.0 | |
| Q1 Exit (V) | 0.0 | 0.0 | | -1.0 | |
| Deflect (V) | -1.6 | -6.6 | | 1.4 | |
| Acquisition mode | Spectrum | | | | |
| Q2 peak pattern | 1 point | | | | |
| Replicates | 3 | | | | |
| Sweeps/replicates | 10 | | | | |
| P/A Pactor Adjustment | yes | | | | |
| Sampler cone / skimmer cone material | Ni-Cu/ Ni | | | | |
| Resolution | unit mass resolution (~1 amu) | | | | |

Table S3: Individual contributions of sample repeatability, calibration curve, the masses of the solution, sample, and standard, and the detector’s drift to the combined relative uncertainty.

|  | Relative uncertainty (u^2(c,r) / Σu^2(c,r) × U) | | | | | | Uncertainty |
| --- | --- | --- | --- | --- | --- | --- | --- |
|  | Repeatability | Calibration curve | Mass (solution) | Mass (sample) | Mass (standard) | Detector's drift | (U(r,c) (k=2)) |
| Ag | 4.34% | 0.50% | 0.00% | 0.01% | 0.02% | 4.03% | 8.9% |
| Al | 2.58% | 11.90% | 0.00% | 0.01% | 0.02% | 3.06% | 17.6% |
| As | 19.22% | 0.26% | 0.00% | 0.00% | 0.01% | 1.28% | 20.8% |
| Ba | 4.74% | 5.14% | 0.00% | 0.01% | 0.02% | 2.75% | 12.7% |
| Ca | 1.61% | 1.15% | 0.00% | 0.02% | 0.02% | 3.18% | 6.0% |
| Cd | 2.64% | 7.24% | 0.00% | 0.01% | 0.02% | 2.92% | 12.8% |
| Co | 2.32% | 0.19% | 0.00% | 0.02% | 0.04% | 2.22% | 4.8% |
| Cr | 4.97% | 22.72% | 0.00% | 0.00% | 0.01% | 0.75% | 28.5% |
| Cu | 1.39% | 0.88% | 0.00% | 0.02% | 0.04% | 3.12% | 5.4% |
| Fe | 1.04% | 1.53% | 0.00% | 0.02% | 0.03% | 4.15% | 6.8% |
| Hg | 1.67% | 7.55% | 0.00% | 0.01% | 0.07% | 2.98% | 12.3% |
| K | 2.24% | 12.60% | 0.00% | 0.01% | 0.01% | 0.13% | 15.0% |
| Mg | 2.91% | 5.45% | 0.00% | 0.01% | 0.02% | 0.26% | 8.6% |
| Mn | 6.83% | 4.56% | 0.00% | 0.01% | 0.02% | 1.36% | 12.8% |
| Mo | 13.78% | 2.72% | 0.00% | 0.01% | 0.02% | 1.28% | 17.8% |
| Na | 4.61% | 7.75% | 0.00% | 0.01% | 0.03% | 0.92% | 13.3% |
| Ni | 8.92% | 9.51% | 0.00% | 0.01% | 0.01% | 1.27% | 19.7% |
| P | 1.13% | 4.25% | 0.00% | 0.02% | 0.03% | 0.47% | 5.9% |
| Pb | 0.96% | 1.41% | 0.00% | 0.01% | 0.03% | 4.66% | 7.1% |
| Rb | 10.70% | 4.52% | 0.00% | 0.01% | 0.01% | 2.02% | 17.3% |
| S | 1.88% | 6.09% | 0.00% | 0.01% | 0.01% | 0.87% | 8.9% |
| Sb | 9.23% | 8.79% | 0.00% | 0.01% | 0.01% | 1.56% | 19.6% |
| Se | 16.56% | 1.10% | 0.00% | 0.01% | 0.01% | 0.89% | 18.6% |
| Sn | 1.58% | 11.09% | 0.00% | 0.01% | 0.01% | 1.99% | 14.7% |
| Sr | 0.72% | 1.27% | 0.00% | 0.02% | 0.03% | 4.20% | 6.2% |
| Ti | 5.40% | 0.16% | 0.00% | 0.01% | 0.03% | 2.03% | 7.6% |
| U | 6.82% | 4.27% | 0.00% | 0.01% | 0.02% | 2.60% | 13.7% |
| V | 5.68% | 6.04% | 0.00% | 0.01% | 0.02% | 1.45% | 13.2% |
| Zn | 3.61% | 0.08% | 0.00% | 0.02% | 0.03% | 2.89% | 6.63% |

Table S4: Results of the method development procedure. Test hair samples obtained from a volunteer were used to evaluate the material of scissors, the appropriate sample dimensions, and the suitability of digestion vessel materials (Teflon, quartz, and glass).  Presented are the mean concentrations (ng/g) and relative standard deviation (%) of quadruplet analyses. Values negative after blank subtraction are excluded from the summary.

| **Scissors** | **Stainless steel** | | | | **Wolfram** | | | |
| --- | --- | --- | --- | --- | --- | --- | --- | --- |
| **Length** | **3 cm** | | **0.2 cm** | | **3 cm** | | **0.2 cm** | |
|  | **mean** | **RSD (%)** | **mean** | **RSD (%)** | **mean** | **RSD (%)** | **mean** | **RSD (%)** |
| **Quartz** | | | | | | | | |
| **Ag** | 362 | 15 | 374 | 15 | 372 | 9 | 348 | 7 |
| **Al** | 5806 | 20 | 6876 | 10 | 6412 | 3 | 6181 | 11 |
| **As** | 9 | 20 | 9 | 17 | 8 | 11 | 8 | 6 |
| **Ba** | 578 | 8 | 689 | 12 | 662 | 9 | 633 | 12 |
| **Ca** | 2.209×10^6 | 9 | 2.675×10^6 | 12 | 2.773×10^6 | 6 | 2.629×10^6 | 11 |
| **Cd** | 63 | 8 | 74 | 15 | 71 | 11 | 67 | 8 |
| **Co** | 130 | 12 | 163 | 14 | 148 | 6 | 279 | 18 |
| **Cr** | 51 | 29 | 238 | 15 | 51 | 9 | 151 | 8 |
| **Cu** | 1.447×10^4 | 14 | 1.588×10^4 | 15 | 1.482×10^4 | 7 | 1.397×10^4 | 7 |
| **Fe** | 8778 | 18 | 1.084×10^4 | 7 | 9889 | 6 | 9261 | 13 |
| **Hg** | 655 | 25 | 631 | 9 | 620 | 8 | 652 | 6 |
| **K** | 1.919×10^4 | 23 | 2.555×10^4 | 22 | 2.429×10^4 | 18 | 2.727×10^4 | 24 |
| **Mg** | 8.922×10^5 | 15 | 1.079×10^6 | 15 | 1.06×10^6 | 6 | 9.761×10^5 | 7 |
| **Mn** | 193 | 24 | 234 | 23 | 341 | 20 | 257 | 32 |
| **Mo** | 36 | 16 | 50 | 16 | 48 | 16 | 36 | 16 |
| **Na** | 1.143×10^5 | 15 | 1.536×10^5 | 18 | 1.142×10^5 | 20 | 1.466×10^5 | 9 |
| **Ni** | 201 | 66 | 205 | 21 | 168 | 31 | 129 | 36 |
| **P** | 9.853×10^4 | 12 | 1.096×10^5 | 9 | 1.099×10^5 | 8 | 1.052×10^5 | 5 |
| **Pb** | 1153 | 24 | 1154 | 14 | 1096 | 8 | 1047 | 7 |
| **Rb** | 24 | 12 | 30 | 10 | 29 | 20 | 27 | 14 |
| **S** | 4.498×10^7 | 11 | 5×10^7 | 13 | 4.824×10^7 | 6 | 4.59×10^7 | 5 |
| **Sb** | 18 | 14 | 18 | 17 | 17 | 11 | 18 | 21 |
| **Se** | 455 | 13 | 527 | 19 | 515 | 10 | 471 | 6 |
| **Sn** | 660 | 10 | 739 | 16 | 719 | 8 | 655 | 10 |
| **Sr** | 8147 | 14 | 9658 | 14 | 9295 | 4 | 8574 | 8 |
| **Ti** | 1.463×10^4 | 12 | 2.111×10^4 | 9 | 1.589×10^4 | 8 | 2.023×10^4 | 6 |
| **U** | 14 | 12 | 17 | 13 | 16 | 6 | 16 | 10 |
| **V** | 31 | 15 | 40 | 10 | 37 | 5 | 34 | 7 |
| **Zn** | 2.292×10^5 | 14 | 2.693×10^5 | 16 | 2.582×10^5 | 5 | 2.475×10^5 | 10 |
| **Teflon** | | | | | | | | |
| **Ag** | 435 | 19 | 320 | 8 | 325 | 16 | 372 | 8 |
| **Al** | 6690 | 14 | 5009 | 8 | 5432 | 7 | 5957 | 14 |
| **As** | 8 | 13 | 8 | 26 | 8 | 4 | 10 | 9 |
| **Ba** | 666 | 10 | 608 | 8 | 583 | 4 | 662 | 11 |
| **Ca** | 2.775×10^6 | 10 | 2.64×10^6 | 5 | 2.491×10^6 | 3 | 2.773×10^6 | 9 |
| **Cd** | 69 | 9 | 61 | 9 | 59 | 10 | 73 | 10 |
| **Co** | 141 | 13 | 137 | 11 | 121 | 9 | 293 | 8 |
| **Cr** | 68 | 57 | 198 | 12 | 40 | 17 | 146 | 12 |
| **Cu** | 1.54×10^4 | 15 | 1.286×10^4 | 6 | 1.292×10^4 | 7 | 1.425×10^4 | 9 |
| **Fe** | 1.027×10^4 | 10 | 8587 | 2 | 8339 | 5 | 9122 | 2 |
| **Hg** | 814 | 24 | 513 | 9 | 491 | 11 | 649 | 8 |
| **K** | 2.521×10^4 | 20 | 2.137×10^4 | 11 | 1.999×10^4 | 10 | 2.658×10^4 | 17 |
| **Mg** | 1.04×10^6 | 11 | 8.757×10^5 | 4 | 9.039×10^5 | 6 | 9.765×10^5 | 11 |
| **Mn** | 224 | 20 | 152 | 6 | 170 | 9 | 207 | 24 |
| **Mo** | 42 | 14 | 43 | 22 | 32 | 6 | 38 | 12 |
| **Na** | 1.108×10^5 | 12 | 1.238×10^5 | 6 | 1.203×10^5 | 7 | 1.433×10^5 | 8 |
| **Ni** | 244 | 36 | 166 | 45 | 134 | 7 | 135 | 13 |
| **P** | 1.172×10^5 | 10 | 1.058×10^5 | 5 | 1.043×10^5 | 4 | 1.126×10^5 | 8 |
| **Pb** | 1126 | 12 | 973 | 5 | 964 | 4 | 1089 | 9 |
| **Rb** | 27 | 25 | 26 | 12 | 25 | 16 | 30 | 12 |
| **S** | 4.881×10^7 | 11 | 4.403×10^7 | 6 | 4.311×10^7 | 3 | 4.765×10^7 | 10 |
| **Sb** | 18 | 12 | 16 | 6 | 16 | 13 | 17 | 10 |
| **Se** | 449 | 15 | 463 | 7 | 436 | 6 | 495 | 6 |
| **Sn** | 690 | 9 | 606 | 9 | 599 | 6 | 674 | 10 |
| **Sr** | 9072 | 11 | 7980 | 8 | 7897 | 9 | 8920 | 9 |
| **Ti** | 2.238×10^4 | 14 | 2.088×10^4 | 10 | 2.04×10^4 | 7 | 2.178×10^4 | 8 |
| **U** | 17 | 17 | 14 | 8 | 14 | 2 | 15 | 10 |
| **V** | 38 | 12 | 32 | 6 | 32 | 9 | 33 | 9 |
| **Zn** | 2.638×10^5 | 13 | 2.248×10^5 | 6 | 2.226×10^5 | 7 | 2.416×10^5 | 9 |
| **Glass** | | | | | | | | |
| **Ag** | 324 | 9 | 329 | 7 | 358 | 20 | 329 | 7 |
| **Al** | 7.315×10^4 | 74 | 5.175×10^4 | 57 | 1.509×10^4 | 230 | 7.069×10^4 | 28 |
| **As** | 10 | 16 | 9 | 17 | 7 | 27 | 9 | 27 |
| **Ba** | 725 | 24 | 658 | 21 | 516 | 14 | 716 | 25 |
| **Ca** | 2.95×10^6 | 15 | 2.899×10^6 | 10 | 2.344×10^6 | 6 | 2.784×10^6 | 2 |
| **Cd** | 64 | 16 | 66 | 10 | 54 | 11 | 64 | 5 |
| **Co** | 140 | 19 | 135 | 10 | 93 | 8 | 269 | 10 |
| **Cr** | -52 | -73 | 84 | 221 | -62 | -15 | -27 | -131 |
| **Cu** | 1.321×10^4 | 14 | 1.305×10^4 | 10 | 1.119×10^4 | 8 | 1.236×10^4 | 1 |
| **Fe** | 1.071×10^4 | 35 | 1.014×10^4 | 14 | 7331 | 20 | 6874 | 9 |
| **Hg** | 618 | 5 | 547 | 10 | 368 | 15 | 557 | 4 |
| **K** | 4.051×10^5 | 81 | 1.616×10^5 | 68 | -1.563×10^4 | -478 | 1.145×10^5 | 70 |
| **Mg** | 9.447×10^5 | 16 | 8.966×10^5 | 8 | 7.512×10^5 | 7 | 8.82×10^5 | 2 |
| **Mn** | 209 | 20 | 202 | 7 | 138 | 13 | 158 | 12 |
| **Mo** | 34 | 71 | 60 | 47 | 5 | 699 | 92 | 123 |
| **Na** | 1.682×10^6 | 90 | 6.952×10^5 | 65 | -4.318×10^5 | -103 | 9.468×10^4 | 140 |
| **Ni** | 182 | 48 | 214 | 64 | 137 | 61 | 187 | 46 |
| **P** | 1.147×10^5 | 13 | 1.087×10^5 | 11 | 9.465×10^4 | 9 | 1.058×10^5 | 3 |
| **Pb** | 1025 | 14 | 1038 | 10 | 943 | 9 | 975 | 2 |
| **Rb** | 293 | 85 | 172 | 51 | 5 | 989 | 110 | 77 |
| **S** | 4.487×10^7 | 10 | 4.38×10^7 | 8 | 3.934×10^7 | 6 | 4.249×10^7 | 2 |
| **Sb** | 16 | 32 | 15 | 6 | 14 | 9 | 16 | 9 |
| **Se** | 402 | 13 | 421 | 1 | 355 | 5 | 353 | 5 |
| **Sn** | 657 | 13 | 664 | 8 | 551 | 11 | 626 | 6 |
| **Sr** | 8705 | 17 | 8625 | 14 | 6776 | 7 | 7915 | 5 |
| **Ti** | 1.586×10^4 | 10 | 1.578×10^4 | 11 | 1.232×10^4 | 10 | 1.557×10^4 | 4 |
| **U** | 16 | 19 | 16 | 13 | 13 | 11 | 15 | 3 |
| **V** | 35 | 17 | 34 | 11 | 30 | 11 | 32 | 2 |
| **Zn** | 2.324×10^5 | 15 | 2.251×10^5 | 10 | 1.991×10^5 | 6 | 2.274×10^5 | 4 |

Table S5: Presented are the standard deviation (SD) and relative standard deviation (RSD) for the unpublished results from an ongoing national HBM study, including 1335 children and adolescents, to which the method is currently being applied.

| **HBM II** | **SD ng/g (RSD %)** |
| --- | --- |
| Ag | 3.58 (257.7) |
| Al | 3.27 (226.9) |
| As | 1.73 (72.8) |
| Ba | 2.57 (156.5) |
| Ca | 1.86 (85.6) |
| Cd | 2.46 (145.9) |
| Co | 3.67 (267.0) |
| Cr | 3.45 (245.5) |
| Cu | 1.53 (53.4) |
| Fe | 1.47 (47.1) |
| Hg | 2.48 (148.3) |
| K | 6.21 (520.9) |
| Mg | 2.16 (115.8) |
| Mn | 2.34 (134.3) |
| Mo | 1.66 (65.9) |
| Na | 4.42 (342.3) |
| Ni | 9.12 (812.1) |
| P | 1.22 (22.1) |
| Pb | 2.80 (179.6) |
| Rb | 5.58 (457.7) |
| S | 1.16 (15.8) |
| Sb | 2.58 (157.8) |
| Se | 1.77 (77.2) |
| Sn | 2.35 (135.1) |
| Sr | 2.91 (191.0) |
| Ti | 2.28 (127.8) |
| U | 3.10 (209.9) |
| V | 2.89 (188.9) |
| Zn | 1.38 (38.5) |

Table S6: Presented are the relative standard deviation (RSD), standard deviation (SD) and recoveries obtained for two certified reference materials (CRMs), IAEA-086 and NIES No. 13, digested in glass, quartz, and Teflon vessels, respectively.

| **CRM** | **IAEA** | | |  | **NIES** | | |
| --- | --- | --- | --- | --- | --- | --- | --- |
| **Vial** | **G** | **Q** | **T** |  | **G** | **Q** | **T** |
| **RSD % (SD ng/g)** | | | | | | | |
| **Ag** | 6 (87.44) | 18 (279.90) | 4 (63.89) |  | 8 (7.93) | 8 (7.14) | 6 (5.16) |
| **Al** | 53 (4.6×10^4) | 10 (6937.74) | 6 (3987.48) |  | 39 (4.013×10^4) | 9 (8349.24) | 6 (5469.59) |
| **As** | 6 (8.03) | 11 (14.16) | 5 (7.02) |  | 7 (6.57) | 7 (6.50) | 2 (2.20) |
| **Ba** | 9 (563.23) | 7 (412.35) | 6 (372.81) |  | 10 (154.11) | 7 (94.43) | 6 (89.48) |
| **Ca** | 14 (7.435×10^4) | 11 (7.807×10^4) | 6 (4.032×10^4) |  | 9 (3.943×10^4) | 11 (5.782×10^4) | 4 (2.089×10^4) |
| **Cd** | 8 (14.59) | 11 (19.83) | 7 (12.34) |  | 8 (20.00) | 7 (17.48) | 5 (11.69) |
| **Co** | 8 (8.62) | 9 (10.46) | 5 (6.50) |  | 9 (6.10) | 10 (7.25) | 9 (6.24) |
| **Cu** | 7 (1240.11) | 8 (1596.17) | 7 (1286.02) |  | 7 (1046.19) | 7 (1072.56) | 4 (606.83) |
| **Fe** | 17 (2.105×10^4) | 11 (1.367×10^4) | 8 (1.06×10^4) |  | 10 (1.251×10^4) | 13 (1.754×10^4) | 9 (1.177×10^4) |
| **Hg** | 3 (12.53) | 7 (41.02) | 3 (13.94) |  | 6 (265.27) | 6 (253.02) | 3 (112.02) |
| **Mg** | 10 (1.903×10^4) | 9 (1.698×10^4) | 5 (9865.82) |  | 8 (1.157×10^4) | 9 (1.329×10^4) | 5 (7401.31) |
| **Mn** | 9 (871.28) | 8 (832.89) | 6 (625.71) |  | 25 (942.01) | 14 (535.18) | 11 (355.59) |
| **Na** | 177 (7.899×10^5) | 10 (7250.34) | 10 (6593.95) |  | 144 (6.448×10^5) | 11 (5150.98) | 7 (3190.98) |
| **Pb** | 7 (767.86) | 7 (843.92) | 4 (513.24) |  | 7 (346.19) | 7 (369.95) | 5 (234.42) |
| **S** | 7 (3.028×10^6) | 8 (3.659×10^6) | 6 (2.571×10^6) |  | 8 (3.473×10^6) | 5 (2.229×10^6) | 4 (1.564×10^6) |
| **Sb** | 16 (8.20) | 8 (4.25) | 9 (5.38) |  | 14 (5.09) | 9 (3.43) | 14 (4.84) |
| **Se** | 11 (112.56) | 10 (110.60) | 9 (88.79) |  | 5 (83.84) | 5 (93.15) | 5 (93.88) |
| **V** | 8 (33.15) | 9 (40.56) | 6 (25.74) |  | 12 (28.20) | 10 (24.32) | 4 (9.66) |
| **Zn** | 9 (1.728×10^4) | 8 (1.602×10^4) | 5 (9485.56) |  | 7 (1.396×10^4) | 7 (1.261×10^4) | 5 (8284.41) |
| **Recovery (%)** | | | | | | | |
| **Ag** |  |  |  |  | 108 | 106 | 101 |
| **Al** |  |  |  |  | 97 | 85 | 83 |
| **As** |  |  |  |  | 103 | 106 | 106 |
| **Ba** |  |  |  |  | 83 | 80 | 82 |
| **Ca** | 111 | 110 | 114 |  | 113 | 104 | 106 |
| **Cd** |  |  |  |  | 126 | 121 | 118 |
| **Co** |  |  |  |  | 112 | 112 | 107 |
| **Cu** | 107 | 118 | 117 |  | 112 | 115 | 108 |
| **Fe** | 108 | 113 | 113 |  | 96 | 110 | 100 |
| **Hg** | 102 | 108 | 111 |  | 110 | 107 | 103 |
| **Mg** | 116 | 121 | 123 |  | 103 | 105 | 99 |
| **Mn** | 116 | 126 | 126 |  | 106 | 110 | 92 |
| **Na** |  |  |  |  | 824 | 85 | 85 |
| **Pb** |  |  |  |  | 123 | 122 | 112 |
| **S** |  |  |  |  | 102 | 101 | 97 |
| **Sb** |  |  |  |  | 100 | 103 | 93 |
| **Se** | 112 | 121 | 111 |  | 115 | 111 | 108 |
| **V** |  |  |  |  | 97 | 98 | 91 |
| **Zn** | 100 | 105 | 106 |  | 101 | 96 | 92 |
